# Short-Term Atrioventricular Dysfunction Recovery after Post-TAVI Pacemaker Implantation

**DOI:** 10.3390/jcdd9100324

**Published:** 2022-09-24

**Authors:** Gaetano Pinnacchio, Eleonora Ruscio, Erica Rocco, Carlo Trani, Francesco Burzotta, Cristina Aurigemma, Enrico Romagnoli, Roberto Scacciavillani, Maria Lucia Narducci, Gianluigi Bencardino, Francesco Perna, Francesco Raffaele Spera, Gianluca Comerci, Antonio Bisignani, Gemma Pelargonio

**Affiliations:** 1 Department of Cardiovascular Sciences, Fondazione Policlinico Universitario Agostino Gemelli IRCCS, Largo Agostino Gemelli 8, 00168 Rome, Italy; 2 Cardiology Institute, Catholic University of Sacred Heart, Largo Francesco Vito 1, 00168 Rome, Italy

**Keywords:** TAVI, permanent pacemaker implantation, AV conduction abnormalities, AV conduction recovery, AV dysfunction after TAVI

## Abstract

Permanent pacemaker implantation (PPI) represents a frequent complication after transcatheter aortic valve implantation (TAVI) due to atrio-ventricular (AV) node injury. Predictors of early AV function recovery were investigated. We analyzed 50 consecutive patients (82 ± 6 years, 58% males, EuroSCORE: 7.8 ± 3.3%, STS mortality score: 5 ± 2.8%). Pacemaker interrogations within 4–6 weeks from PPI were performed to collect data on AV conduction. The most common indication of PPI was persistent third-degree (44%)/high-degree (20%) AV block/atrial fibrillation (AF) with slow ventricular conduction (16%) after TAVI. At follow-up, 13 patients (26%) recovered AV conduction (i.e., sinus rhythm with stable 1:1 AV conduction/AF with a mean ventricular response >50 bpm, associated with a long-term ventricular pacing percentage < 5%). At multivariate analysis, complete atrio-ventricular block independently predicted pacemaker dependency at follow-up (*p* = 0.019). Patients with persistent AV dysfunction showed a significant AV conduction time prolongation after TAVI (PR interval from 207 ± 50 to 230 ± 51, *p* = 0.02; QRS interval from 124 ± 23 to 147 ± 16, *p* < 0.01) compared to patients with recovery, in whom AV conduction parameters remained unchanged. Several patients receiving PPI after TAVI have recovery of AV conduction within a few weeks. Longer observation periods prior to PPI might be justified, and algorithms to minimize ventricular pacing should be utilized whenever possible.

## 1. Introduction

Aortic stenosis is the most common primary valve disease in developed countries, with a rapidly increasing prevalence due to the aging population [1]. Over the last decade, transcatheter aortic valve implantation (TAVI) has become a valuable option for surgical aortic valve replacement (SAVR) in a wider spectrum of patients with severe symptomatic aortic stenosis (AS) [2]. As a result, the number of related complications has correspondingly augmented, variably affecting long-term outcomes [3]. In particular, conduction abnormalities (CAs) requiring permanent pacemaker implantation (PPI) represent a frequent complication after TAVI, approximately ranging between 3 and 26% in randomized clinical trials and large registries [4]. Several predictors for PPI after TAVI have been identified, including both patient and procedural factors [5]. Among the putative mechanisms, anatomical proximity to the aortic root has been possibly linked to iatrogenic injury of the AV conduction system [6]. Recent studies have shown that a significant number of patients are not strictly pacemaker-dependent over time, meaning that AV CAs may recover, thus making unnecessary pacemaker therapy in the long term, together with its potential complications [7,8]. Nevertheless, data on the features and time course of a possible AV conduction restoration during the post-operative period after TAVI are still lacking [5,9]. The aim of our study was to investigate the prevalence and predictors of early CAs recovery in patients who underwent PPI due to AV node dysfunction after TAVI.

## 2. Materials and Methods

### 2.1. Patient Selection

A total of 392 consecutive patients eligible for the study, who underwent TAVI at our Department were screened. From these, 25 patients with a pre-existing pacemaker were excluded. Of the 392 patients, 102 (26%) developed new AVB of various degrees post-TAVI. Of these, 52 showed a stable resolution/improvement of the AV disfunction and were not considered eligible for definite pacemaker implantation, while the remaining 50 patients showed a persistence of a HAVB (i.e., paroxysmal or persistent) with indications for permanent pacemakers. The indication for TAVI was made by a multidisciplinary Heart Team consisting of cardiologists, cardiac surgeons, and anesthetists, in accordance with current guidelines after extensive invasive and non-invasive examinations including echocardiography, computed tomography for the study of the aortic valve and vascular access, coronary angiography/coronary CT [10]. PPI was performed after TAVI due to intermittent/paroxysmal or new continuous high degree AVB (HAVB)/complete AVB (CAVB), AVB 2nd degree Mobitz 2, AVB 2:1, and atrial fibrillation with slow ventricular conduction (defined as a sustained heart rate less than 50 bpm), in accordance with local practice and as provided by guidelines/consensus papers [4,11,12].

### 2.2. Baseline Data

All patients underwent the same clinical and instrumental workup. Baseline features including age, gender, cardiovascular risk factors, history of cardiovascular disease, and medical therapy were collected. Continuous ECG monitoring was performed via telemetry, and a standard 12-lead ECG was acquired before and immediately after TAVI and every 24 h until PPI, to evaluate rhythm, mean heart rate, PR, QRS, and QT intervals duration, presence of intraventricular conduction disturbances. Electrocardiographic intervals were measured using digitized tracings with calipers allowing 1-millisecond resolution at a screen velocity of 200 mm/s. A complete transthoracic echocardiographic examination was performed on all patients by an experienced blinded sonographer, using the commercially available system Toshiba Artida with a 3.5 MHz phased array transducer, to obtain preoperative both qualitative and quantitative imaging data, according to recommendations [12]. A computer tomography (CT) scan was performed to evaluate the preferable vascular site of access for the TAVI (transfemoral, transapical, or transaortic) and to access the correct type and size of the prosthesis; finally, all patients underwent cardiac CT or coronary angiography to document coronary anatomy. In case of significant coronary stenosis, patients were evaluated for PCI prior to TAVI. The study protocol was approved by our institutional ethic committee. Informed consent was obtained from all subjects involved in the study.

### 2.3. TAVI Procedure

TAVI procedures were performed under anesthesia in our hybrid operating room. Negative dromotropic medications such as betablockers, non-dihydropyridine calcium channel blockers, or digoxin were stopped 1–2 days before TAVI. The access was previously guided by the CT scan data: usually, a percutaneous transfemoral approach was preferred but also a ventricular transapical or transaortic approach was performed in case of the calcified or inadequate peripherical vascular route. A temporary ventricular pacemaker allowed an adequate positioning of both balloon-expandable and self-expandable aortic valve prosthesis, through high-frequency pacing to reduce cardiac output. Balloon aortic valvuloplasty was performed if deemed required by the operator. Postoperatively, continuous ECG monitoring was performed via telemetry, and a standard 12-lead ECG was acquired daily.

### 2.4. PPI and Follow-Up

All patients received pacemakers during their stay in the hospital. Timing to PPI was dependent on the presentation of the bradyarrhythmia as well as on the rate of ventricular escape rhythm, based on the surgeon’s assessment. Pacemakers were programmed according to the underlying rhythm and standardized in all patients. At the time of PPI, single-chamber PMs were programmed to a VVIR mode and dual-chamber PMs to the DDD(R)-mode, activating proprietary algorithms in order to minimize ventricular pacing, whenever applicable. In particular, the stimulation mode was programmed in the DDD-mode without ventricular intrinsic preference (VIP)/AV delay hysteresis in patients showing persistent second or third-degree AVB as well as first-degree AVB with a PQ time exceeding 400 ms, or in the VVIR-mode in those with atrial fibrillation and low ventricular conduction, whereas in all other cases the device-specific VIP program was activated in order to promote spontaneous ventricular activation as much as possible. The lower rate limit was set to 50–60 beats per minute (bpm) in all devices. At pre-discharge, a further PM interrogation was performed in all patients. In cases of right ventricle stimulation, VVI mode 30 bpm was temporarily programmed to check the native AV conduction. If appropriate, in the presence of intrinsic 1:1 AV conduction, the pacing mode was re-programmed in order to limit ventricle stimulation and favor spontaneous ventricular activation (e.g., AV interval was set up to the maximum value possible). All patients underwent at least a new device interrogation 4 weeks after implantation. All interrogations were reviewed and coded to include the percentage of time paced in the atria and ventricles, underlying AV conduction and rhythm, and pacemaker dependency. Right ventricular pacing percentage (VPP) was determined by values reported on device interrogation. Based on the results of pacemaker interrogations, patients were divided into two groups according to the recovery of AV conduction at follow-up. Effective restoration of AV conduction was defined as sinus rhythm with 1:1 AV conduction or atrial fibrillation with a mean ventricular response >50 bpm, in association with a VPP <5% in the last weeks documented throughout the statistics processed by the device. We made the choice to include only patients with a poor long-term VPP in the AV function recovery group to exclude subjects with temporary AV conduction recovery (e.g., evidence of 1:1 AV conduction restoring at the time of the first pacemaker interrogation) but possible episodes of paroxysmal AV node dysfunction requiring pacing by the pacemaker (also considering that the device was programmed in order to promote spontaneous rhythm).

### 2.5. Statistical Analysis

Continuous variables are expressed as mean ± standard deviation or by median (interquartile range). Categorical differences between groups were carried out using Pearson’s chi-square test, while an unpaired Student’s t-test or the Mann–Whitney U test were used to compare differences between means. Binary logistic regression analysis was performed to assess the association of baseline clinical variables with the recovery of AV conduction. A *p* value < 0.05 was considered significant for all statistical determination. Analyses were performed using SPSS software version 20.0 (IBM Corporation, Armonk, New York, NY, USA).

## 3. Results

### 3.1. Baseline Findings

The baseline clinical characteristics of the study population are summarized in Table 1.

The whole population (50 patients; 82 ± 6 years, 58% males) was a high-risk group for cardiac surgery, as assessed by EuroSCORE (7.8 ± 3.3%) and STS mortality score (5 ± 2.8%) with concomitant multi-morbidity. Here, 21 (42%) patients had a previous diagnosis of ischemic cardiac disease. Eight (16%) patients had persistent atrial flutter/fibrillation. A preexisting bundle branch block was reported in eight (16%) patients, three (6%) of them showing a left bundle branch block (LBBB) and five (10%) a right bundle block (RBBB).

Most aortic valve implantations were performed electively (42 patients, 84%), with transfemoral access used as the preferred approach (94%). The prevalent indication for TAVI was severe aortic stenosis of the native valve. One patient had a previous surgical aortic valve replacement. The self-expandable prosthesis was chosen in a greater extent (94% of cases). No major complications related to TAVI occurred; a severe paravalvular leak was documented in 2 (4%) patients.

### 3.2. Indication for Permanent Pacemaker Implantation and Device Programming

All patients received pacemakers during the same hospitalization for TAVI. The most common indication for pacemaker implantation was persistent third-degree AVB (22 patients, 44%). The other indications for pacemaker implantation were: persistent HAVB (10 patients, 20%); atrial fibrillation with slow ventricular conduction (8 patients, 16%); intermittent HAVB with new LBBB (6 patients, 12%); intermittent HAVB without new LBBB (4 patients, 8%). Therefore, 37 patients (74%) presented with permanent arrhythmia leading to PPI, while 13 (26%) presented with paroxysmal rhythm disturbance. Out of the total 50 patients, 35 required temporary pacemaker implantation. The delays between the TAVI procedure and PPI ranged from 1 to 10 days (median 4.5 days, interquartile range-IQR: 2–6 days). The majority of patients (35/50, 70%) implanted pacemakers within 6 days post-procedure.

### 3.3. Recovery of Conduction

At the first pacemaker interrogation (i.e., 4 weeks after implantation), patients were classified into two groups, on the basis of AV conduction recovery: 13 patients (26%) showed sinus rhythm with 1:1 AV conduction (or atrial fibrillation with a mean ventricular response > 50 bpm), in association with a VPP < 5%, and were assigned to Group 1; the other 37 patients (74%), without recovery of AV conduction or with AV conduction recovery but with a VPP > 5% despite adequate pacemaker programming, were assigned to Group 2.

The main clinical and ECG findings of Group 1 and Group 2 patients are summarized in Table 2. Notably, “TAVI-to-PPI time” was significantly higher in patients showing AV conduction recovery compared to patients with persistence of AV dysfunction. At ECG pre-TAVI, no significant differences were found between groups with respect to AV conduction time and prevalence of typical bundle branch block, while mean QRS width was significantly longer in patients of Group 2 compared to those of Group 1 (*p* = 0.018). There was a lower rate of conduction recovery in patients with pacemakers placed for persistent third-degree AVB compared to those implanted for other indications (13% vs. 39%, *p* = 0.03, respectively).

In addition, in 22 patients the measure of PR interval after TAVI was obtained (i.e., excluding 28 patients who developed persistent AVB post-TAVI other than first-degree AVB and patients with atrial fibrillation). Analyzing ECG data in these subgroups of patients, a significant lengthening of both atrioventricular and intraventricular conduction time after TAVI was found only in Group 2 (from 207 ± 50 to 230 ± 51 ms, *p* = 0.02; and from 124 ± 23 to 147 ± 16 ms, *p* < 0.01, respectively), whereas in Group 1 PR interval remained substantially unchanged (from 198 ± 36 to 200 ± 35 ms, *p* = 0.31) and QRS showed only a mild, not statistically significant, increment (from 107 ± 14 to 127 ± 24 ms, *p* = 0.09).

**Figure 1:**
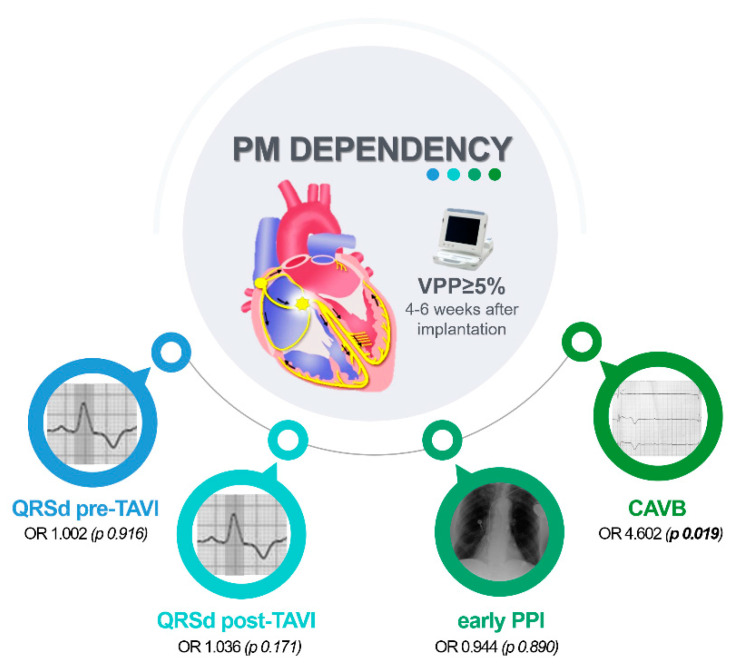
Pacemaker dependency associated factors. At univariate analysis, the variables statistically associated with pacemaker dependency after TAVI (ventricular pacing percentage VPP ≥ 5% 4–6 weeks after PPI) were: QRS duration (QRSd) before TAVI, QRSd after TAVI, early PPI after TAVI and occurrence of CAVB. At multivariate analysis, only the occurrence of CAVB independently predicted pacemaker dependency at follow-up (*p* = 0.19; see text for details).

The variables associated with pacemaker dependency at univariate analysis were: prolonged QRS duration (QRSd) before TAVI, prolonged QRSd after TAVI, early PPI after TAVI, and occurrence of CAVB (Table 3). Notably, the analysis of the association between RBBB and the outcome was not performed due to all patients with RBBB experiencing the event. Finally, at multivariate analysis, only the occurrence of CAVB independently predicted pacemaker dependency at follow-up (Table 3, Figure 1).

## 4. Discussion

The need for pacemaker implantation after TAVI seems to remain an unresolved issue, due to limited available data. Several patient- and procedure-related factors have been associated with PPI after TAVI, including advanced age, male gender, atrial fibrillation, calcification of aortic and mitral annulus, small LVOT, pre-existence of a RBBB or intraprocedural CAs, balloon pre-dilation, valve type and depth of prosthesis implantation [5].

Accordingly, the purpose of our study was to better quantify the rate and the predictors of conduction recovery in patients with advanced AV dysfunction who require pacemaker implantation after TAVI.

Although the association between pacing after TAVI and outcome is still controversial [3,13,14], long-term right VP may cause electromechanical asynchrony, negative left-ventricular remodeling, and increased risk for atrial fibrillation, leading to heart failure [14,15]. Hence, PPI after TAVI has been linked to increased length of intensive care unit, hospital stay, and post-procedural costs [13]. In this regard, current guidelines recommend a more conservative approach under watchful waiting after TAVI [4], particularly in those categories of patients who may present temporary CAs liable to recover over time [5,8,16].

A recent, large study [17] analyzed the predictors of third-degree AVB persistence with concurrent permanent PM dependency (defined as VP ≥ 5%) after TAVI, concluding that it may be wise to postpone the indication for PPI as AVB generally solves. Indeed, CAs after TAVI have been partially related to local edema and inflammation of the injured tissue, due to mechanical pressure of the implanted valve on the atrioventricular bundle, and thus may heal [18].

Moreover, a retrospective analysis [19] evaluated the incidence of HAVB after TAVI and the percentage of VP and PM dependency at 6–8 weeks after implantation and concluded that more than half of the patients were not strictly PM-dependent, but presented an underlying intrinsic rhythm.

Additionally, Marzahn et al. investigated AV node conduction recovery in a wide population of TAVI patients, finding that 45% of them showed sufficient AV node conduction after pacemaker reprogramming at follow-up [16].

In our study, we found that 25% of patients who received pacemakers after TAVI had restored AV conduction, such as demonstrated in device interrogations within the 4–6 weeks after pacemaker implantation. Therefore, our results seem to be consistent with previous studies, most of which examined relatively small cohorts, as ours, and with an average of less than one year of follow-ups. In those studies, recovery of AV node conduction was found in 50–73% of patients [13,20], thus superior to the rate observed in our population. Most studies defined PM non-dependency as VP < 5%, as we straightforwardly did [21]. It is worth mentioning that the percentage of VP may generally be influenced by different factors, including increased overnight pacing and “out of the box”, standardized algorithms which threaten tailored ones. Given the low rates of long-term PM dependency, efforts to minimize unnecessary permanent pacing are logically warranted [4] and may be therefore instituted if conduction recovers.

The present study adds to known literature by specifically examining the AV conduction restoring through ECG post-TAVI and pacemaker interrogations. Our sample size was powered to find some predictors of recovery, including a shorter width of the QRS both before and after the TAVI, pacemakers placed for indications other than third-degree AVB, and implanted later after TAVI. In fact, current guidelines [4] propose standard management of patients with persistent HAVB that persists for 24–48 h after TAVI or new onset alternating AVB, whether patients with other CAs at baseline or post-procedure should undergo a more cautious treatment. Of notice, our patients reporting CAVB witnessed a condition of PM-dependency at follow-up. Hence, our study enriches the set of evidence in the pipeline. However, it is not clear why reliable predictors of long-term pacing have not been identified. As previously stated, recovery of conduction may be possibly due to the variable anatomic relationships of how close the left bundle runs in relation to the MS and endocardium. In addition, the deeper extent of some devices (e.g., Medtronic CoreValve) into the conduction system and the augmenting radial force from self-expansion complicate both the timing of the original implant as well as prediction of the possibility or timing of recovery, generating further hypothesis.

Nevertheless, a meta-analysis has even found a trend toward a protective effect from cardiac death in the first year after the procedure. One possible explanation for this finding may be that PPI may protect patients with conduction disorders from potential progression toward complete AVB. For instance, Auffret et al. recorded a pre-existing RBBB in about 10% of TAVI recipients. Patients with an RBBB without a PPM at hospital discharge may be at especially high risk for HAVB and sudden cardiac death during follow-up [22]. Unfortunately, we could not analyze the predictive value of baseline RBBB on the outcome as no one RBBB patient in our sample restored normal conduction.

In accordance with up-to-date literature, the results of our study place in the mainstream reconsidering the observation period prior to pacemaker placement for HAVB after TAVI. In practical terms, longer periods of observation need to be balanced against the disadvantages of prolonged temporary pacing (i.e., risk of infections); the currently proposed duration of observation has been agreed upon by consensus of experts, far from being properly determined.

Further research is needed on the long-term outcomes of AV conduction after TAVI. The next steps should include instituting a prospective multicenter study design that would allow for uniform device programming and protocoled interrogations at set times post PPI to more exactly approximate the timing of AV conduction recovery. As the potential pool of candidates for TAVI expands from high-risk surgical candidates to a larger population, determinations about how long to observe these patients post-TAVI and in whom to place a permanent pacemaker become even more important.

The most significant limitation of our study is a relatively small sample size. Furthermore, our study is limited by its retrospective design. Nonetheless, a relatively long time of recruitment was handled with the implementation of the TAVI procedure over time, while maintaining adherence to updated clinical practice. The timing and number of follow-up device interrogations were at the discretion of the clinicians and patients’ compliance with recommended follow-up. It should be noted that recovery of conduction does not necessarily imply that pacing could be avoided, as some patients might still have profound first-degree AV block or sinus bradycardia to take care of. Based on a physiopathological rationale of healing from acute tissue injury and in accord with previous studies in the pipeline, we explored predictors of early conduction recovery. Further validation of this model is required. Finally, we used ventricular pacemaker stimulation at the time of interrogation at a single point in time; we were unable to clearly define whether or not these patients intermittently had HAVB.

## 5. Conclusions

This retrospective study found that several patients who received pacemakers for AV conduction disturbances post-TAVI have recovery of conduction. Since many patients restore conduction within weeks of pacemaker implantation, longer observation periods prior to PPI might be justified, and algorithms to minimize ventricular pacing should be utilized whenever possible.

## Figures and Tables

**Table 1:** Main clinical, ECG and procedural characteristics of patients included in the study.

Variables	All Population (*n* = 50)
*Clinical characteristics*	
Age, y, mean ± SD	82 ± 6
Male, n (%)	29 (58)
BMI, kg/m^2^, mean ± SD	27 ± 5
Hypertension, n (%)	44 (88)
Diabetes, n (%)	14 (28)
Smokers, n (%)	4 (8)
Dyslipidemia, n (%)	28 (56)
Familiar history of CVD, n (%)	12 (24)
Obesity, n (%)	12 (24)
Peripheral artery disease, n (%)	9 (18)
Chronic renal diseases, n (%)	18 (36)
NYHA class III-IV, n (%)	36 (72)
Coronary artery diseases, n (%)	21 (42)
Euroscore, %, mean ± SD	7.8 ± 3.3
STS score mortality, %, mean ± SD	5 ± 2.8
*ECG characteristics*	
ECG-PR, ms, mean ± SD	203 ± 52
ECG-QRS, ms, mean ± SD	118 ± 23
ECG-QT, ms, mean ± SD	415 ± 36
Atrial Fibrillation, n (%)	8 (16)
ECG-BBS, n (%)	3 (6)
ECG-BBD, n (%)	5 (10)
*Procedural characteristics*	
Elective, n (%)	42 (84)
Delay from TAVI and PM implantation, d, mean ± SD	6 ± 6
Valvolar CT area, mm2, mean ± SD	420 ± 51
Apical Approach, n (%)	3 (6)
Valve in valve, n (%)	1 (2)
Bicuspid native valve, n (%)	1 (2)
TAVI balloon expandable, n (%)	3 (6)
Pre-TAVI aortic systolic blood pressure, mmHg, mean ± SD	142 ± 17
Post-TAVI aortic systolic blood pressure, mmHg, mean ± SD	163 ± 20
Severe post-TAVI aortic regurgitation	2 (4)

BMI: Body Mass Index; CVD: cardiovascular diseases; NYHA class: New York Heart Association class; STS: Society of Thoracic Surgeons.

**Table 2:** Clinical and ECG findings in patients with low (<5%, Group 1) and high (≥5%, Group 2) ventricular pacing percentage.

Variables	Group 1 (*n* = 13)	Group 2 (*n* = 37)	*p*-value
**Age, y, mean ± SD**	83 ± 5	81 ± 7	0.71
**Male, n (%)**	9 (64)	20 (55)	0.75
**BMI, kg/m^2^, mean ± SD**	26 ± 6	28 ± 3	0.47
**Hypertension, n (%)**	12 (92)	32 (89)	1.00
**Diabetes, n (%)**	6 (46)	8 (23)	0.16
**Smokers, n (%)**	0 (0)	4 (11)	0.56
**Dyslipidemia, n (%)**	7 (54)	21 (58)	1.00
**Familiar history of CVD, n (%)**	1 (8)	11 (31)	0.14
**Obesity, n (%)**	2 (15)	10 (28)	0.47
**Peripheral artery disease, n (%)**	2 (15)	7 (19)	1.00
**Chronic renal disease, n (%)**	6 (46)	12 (33)	0.51
**NYHA class III-IV, n (%)**	9 (69)	27 (75)	0.72
**Coronary artery diseases, n (%)**	4 (33)	17 (47)	0.51
**Euroscore, %, mean ± SD**	8.2 ± 3.5	7.7 ± 3.2	0.62
**STS mortality, %, mean ± SD**	4.9 ± 2.4	5.0 ± 3.0	0.91
**TAVI-to-PPI-time, days, mean ± SD**	9 ± 6	5 ± 5	**0.03**
**PR pre-TAVI,ms, mean ± SD**	198 ± 36	207 ± 50	0.78
**PR post-TAVI,ms, mean ± SD**	200 ± 35	230 ± 51	0.16
**QRS pre-TAVI,ms, mean ± SD**	107 ± 14	124 ± 23	**0.018**
**QRS post-TAVI,ms, mean ± SD**	127 ± 24	147 ± 16	**0.018**
**QT pre-TAVI,ms, mean ± SD**	403 ± 35	420 ± 36	0.28
**QT post-TAVI,ms, mean ± SD**	430 ± 54	462 ± 59	0.27
**Atrial Fibrillation, n (%)**	3 (21)	5 (14)	0.54
**ECG-LBBB, n (%)**	7 (19)	4 (29)	0.25
**ECG-RBBB, n (%)**	0 (0)	6 (17)	0.11

Bold values denote statistical significance (see text).

**Table 3:** Predictors of high PM ventricular stimulation (VPP ≥ 5%).

Variables	*Univariate Analysis*			*Multivariate Analysis*		
	OR	CI 95%	*p*	OR	CI 95%	*p*
**PR Pre-TAVI**	0.989	0.890–1.100	0.844			
**QRS Pre-TAVI**	1.039	1.004–1.076	**0.029**	1.002	0.969–1.036	0.916
**Age**	0.989	0.890–1.100	0.844			
**Euroscore**	0.966	0.796–1.172	0.724			
**TAVI-PM Implant (days)**	0.882	0.774–1.006	**0.061**	0.944	0.845–1.215	0.890
**AF**	0.632	0.129–3.102	0.572			
**LBBB**	2.750	0.524–14.439	0.232			
**LBBB post**	0.865	0.217–3.458	0.838			
**PR post-TAVI**	1.018	0.993–1.044	0.168			
**QRS post-TAVI**	1.054	1.004–1.107	**0.034**	1.036	0.985–1.090	0.171
**Complete AVB**	4.354	1.032–18.367	**0.045**	4.602	1.280–9.408	**0.019**

Bold values denote statistical significance (see text).

## Data Availability

Data are available from the authors upon appropriate request of the Editor.
